# A Graph Theory approach to assess nature’s contribution to people at a global scale

**DOI:** 10.1038/s41598-021-88745-z

**Published:** 2021-04-27

**Authors:** Silvia de Juan, Andrés Ospina-Álvarez, Sebastián Villasante, Ana Ruiz-Frau

**Affiliations:** 1 Department of Renewable Marine Resources, Institute of Marine Science (ICM-CSIC), Passeig Marítim de la Barceloneta, n° 37-49, 08003 Barcelona, Spain; 2 Department of Marine Ecosystem Dynamics, IMEDEA (CSIC-UIB), Miquel Marqués 21, 07190 Esporles, Spain; 3 Faculty of Economics and Business Administration, University of Santiago de Compostela, Av. Burgo das Nacións s/n, 15782 Santiago de Compostela, A Coruña Spain; 4 Campus Do Mar, International Campus of Excellence, Campus Universitario, 36310 Vigo, Spain

**Keywords:** Sustainability, Ecosystem services

## Abstract

The use of Graph Theory on social media data is a promising approach to identify emergent properties of the complex physical and cognitive interactions that occur between humans and nature. To test the effectivity of this approach at global scales, Instagram posts from fourteen natural areas were selected to analyse the emergent discourse around these areas. The fourteen areas, known to provide key recreational, educational and heritage values, were investigated with different centrality metrics to test the ability of Graph Theory to identify variability in ecosystem social perceptions and use. Instagram data (i.e., hashtags associated to photos) was analysed with network centrality measures to characterise properties of the connections between words posted by social media users. With this approach, the emergent properties of networks of hashtags were explored to characterise visitors’ preferences (e.g., cultural heritage or nature appreciation), activities (e.g., diving or hiking), preferred habitats and species (e.g., forest, beach, penguins), and feelings (e.g., happiness or place identity). Network analysis on Instagram hashtags allowed delineating the users’ discourse around a natural area, which provides crucial information for effective management of popular natural spaces for people.

## Introduction

Marine and coastal areas are extremely important for human wellbeing and yet, management plans rarely consider in their formulation the contribution of these natural areas to society^1,2^. The assessment of the contribution of nature to people wellbeing is particularly challenging due to its intangible and subjective nature^3,4^. Research targeted at marine ecosystem services has mostly focused on the economic valuation of commercial fishing, recreational activities, tourism, or seascape scenic beauty^5,6^, setting aside the non-material benefits people obtain from ecosystems that have symbolic, cultural or intellectual significance^7^. During the past decade, research on marine conservation has evolved to acknowledge the importance of the non-use value of ecosystems^8^ and it currently needs to be incorporated in conservation and management plans^9^. However, the incorporation of a standard assessment of non-material benefits of ecosystems in conservation and management is challenging because, among others, assessments have been often based on time consuming field survey methods^10^, including interviews, face-to-face questionnaires and participatory mapping^11–14^. These assessments are often conducted at a site-scale^15^, whereas conservation plans generally need information on the interaction between humans and ecosystems at multiple spatial scales^16^. In this context, new cost-effective methodological approaches are needed to be implemented at scales larger than the local case study. There are several studies that adopt large scale approaches^2,17^, but these are not cost-effective for a generalized implementation.

In the internet era, there are many social network platforms with millions of users that are an important source of big data. These platforms continuously store information people upload from any location on the planet, as they are used for socializing and communicating. Importantly, social media content is frequently related with recreational activities, including tourism^18^. As part of the information uploaded, people often express their feelings about natural spaces^19^. In the quest to avoid the time-consuming nature of field surveys and to identify alternative methods, there has been an increasing number of scientific studies that use social media for an indirect assessment of peoples’ perceptions and preferences^10,18,20^. These studies have generally proved to be comparable to traditional surveys^21^. The use of social media data initially relied on photo content assessment (but see Geboers et al.^22^). The context and content of the photographs is classified into cultural ecosystem services’ categories based on the presence or absence of specific elements in the photos, such as views of flora and fauna, historical buildings, or touristic infrastructure and facilities^23^. During the past decade, scientific works have also explored different analytical techniques on the text associated to the images, including sentiment analysis, Natural Language Processing and data mining methods^24–26^. Recent developments in the analysis of social media data have also applied Graph Theory to hashtags associated to posts, providing a promising approach for the remote assessment of Cultural Ecosystem Services^27^. However, the application of this approach has been so far limited to the regional scale, while it offers an untapped potential to be applied at a global scale and to provide comparative information on the type of marine and coastal ecosystem contribution to societies around the world.

Instagram social media is generally used to post photographs and thoughts in real-time often related to activities or social recreation, but also to cultural and wildlife appreciation^27^. An advantage associated to Instagram is the frequent inclusion of hashtags as part of the photo post. These hashtags are used as keywords to mark messages or form conversations and, thus, they provide an additional way to connect visual content (i.e., photos) and semantically related words to a discourse. User-generated hashtags provide an opportunity to analyse the discourse linked to the photos and minimize the subjectivity and low-cost effectiveness associated to photo content analysis.

On the other hand, Graph Theory, as the mathematical study of the interaction of a system of connected elements, is a suitable approach for analysing the string of words associated to an Instagram post. The analysis of networks using Graph Theory can be described as the analysis of existing relationships between the different elements contained in a network. It provides a simplified and quantitative view of the multiple factors involved in the connection among system elements^28^. The term *vertex*, or node, is used to describe the elements in a network, while the term *edge* is used to refer to the connections between the different vertices in a network. In our case, vertices are represented by hashtags, while edges illustrate the connections between hashtags. In a network of keywords posted with the photos, Graph Theory provides insights into the system properties and identifies critical nodes with high centrality (i.e., words connected to many other words) or clusters of well-connected nodes^29–31^.

**Figure 1:**
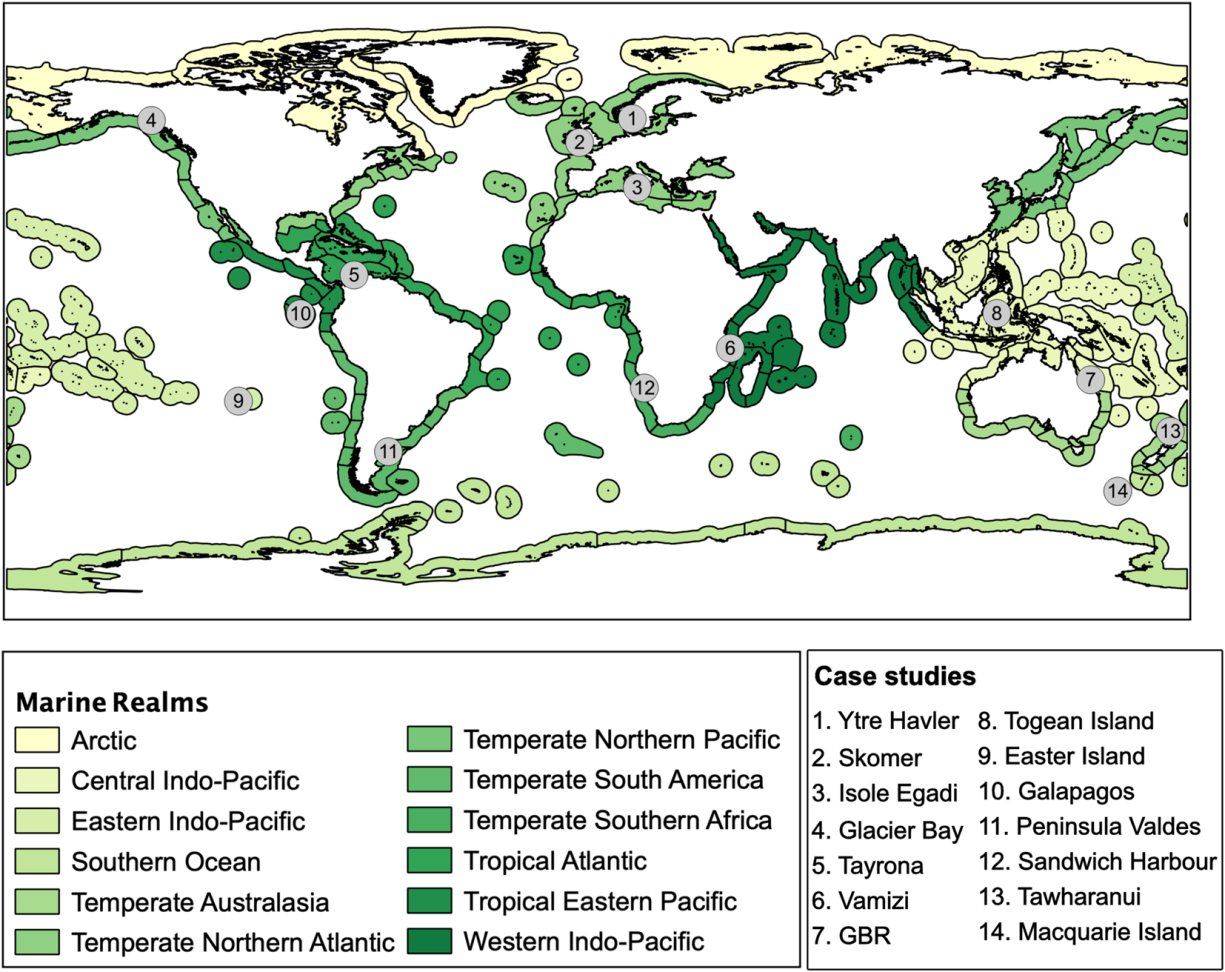
The 14 case studies selected across the twelve marine realms proposed by Spalding et al.^32^.

In this study, the working hypothesis was that data extracted from Instagram and analysed through different Graph Theory centrality measures can be used to understand peoples’ preferences for nature and nature-based experiences in marine and coastal areas worldwide, regardless of their geographical location, environmental characteristics, accessibility, tourism features, or any differentiating characteristics. The hypothesis was tested in 14 marine and coastal areas that span over the 12 marine realms established by Spalding^32^ (Fig. 1 and Table 1). These areas are expected to provide a wide diversity of benefits to society (e.g., recreation, cultural heritage, nature and wildlife observation) and to be visited by a wide diversity of users. This approach has the potential to contribute to cost-efficient assessments of the contribution of marine and coastal areas to society well-being.

**Table 1:** Case study name, location, query and number of posts downloaded for the study.

Case study	Location	Query	Number of posts
Galapagos	Ecuador	#galapagos	10,000
Glacier Bay	Alaska	#glacierbayalaska	1811
Great Barrier Reef	Australia	#greatbarrierreef	9960
Isole Egadi	Italy	#isoleegadi	9969
Macquarie Island	Australia	#macquarieisland	1430
Peninsula Valdez	Argentina	#peninsulavaldes	9971
Easter Island	Chile	#easterisland, #rapanui, #isladepascua	10,000
Sandwich Harbour	Namibia	#sandwichharbour	2807
Skomer	United Kingdom	#skomer	4911
Tawharanui	New Zealand	#tawharanui	6832
Tayrona	Colombia	#tayrona	10,000
Togean Island	Indonesia	#togeanisland	9467
Vamizi	Mozambique	#vamizi	1367
Ytrehvaler	Norway	#ytrehvalernasjonalpark	1019

## Results

For each case study area, a search query was executed (Table 1). Query terms were based on the hashtags of the geographical name of the study areas; therefore, the post download was related to the name of the study area (e.g., Galapagos), with all downloaded posts including this name as query. Query search was limited to English, the most common language amongst tourists. This might have overlooked posts where the name of the place was in a different language. For most marine areas, this was considered irrelevant as the name of the place is not translated to other languages (e.g., Tayrona, Vamizi, Skomer). In some of the cases, the name of the place could appear in a variety of languages (e.g., Great Barrier Reef), however, the use of non-English place hashtags as queries generally retrieved a significantly lower number of posts (e.g., Gran Barrera de Coral in Spanish with 1900 posts, or Grand Barrière de Corail in French with 14 posts, while Great Barrier Reef had over 10,000 posts). In the specific case of Easter Island, we observed that the use of three particular queries was linked to a high number of posts: Easter Island and the local name Rapanui had over 10,000 posts each, and Isla de Pascua in Spanish had 8700 posts. In this case, three separate posts’ downloads were performed, and data were merged for subsequent analysis. The above, rather than a limitation of the methodological approach, demonstrates its flexibility to adapt to different data acquisition requirements.

To illustrate the most relevant information contained as part of the posts downloaded for each of the 14 areas, we selected the 150 most frequent hashtags from each dataset in order to create the network graph and represent the dominant discourse in relation to the area in question. Network graphs were delineated using eigenvector, betweenness and edge betweenness as centrality measures. Eigenvector centrality measure (hereafter Eigenvector) allows identifying those hashtags that are frequently posted with other hashtags also frequently posted, and it can be interpreted as the pairs or groups of features more frequently related to the case study by the users. Betweenness centrality (hereafter betweenness) and edge betweenness centrality (hereafter edge betweenness) provide information about clusters of hashtags that describe users' experiences or perceptions and that connect (by means of a hashtag) to other clusters representing other types of experiences or perceptions. These high betweenness hashtags structure the general discourse about an area and their removal would fragment the network and disconnect distant concepts. Therefore, hashtags and links with high betweenness can show the discourse parallel or additional to the main discourse and their relations, allowing to identify less frequent activities or perceptions but that are equally important to understand the network as a whole.

### Network centrality measures

**Figure 2:**
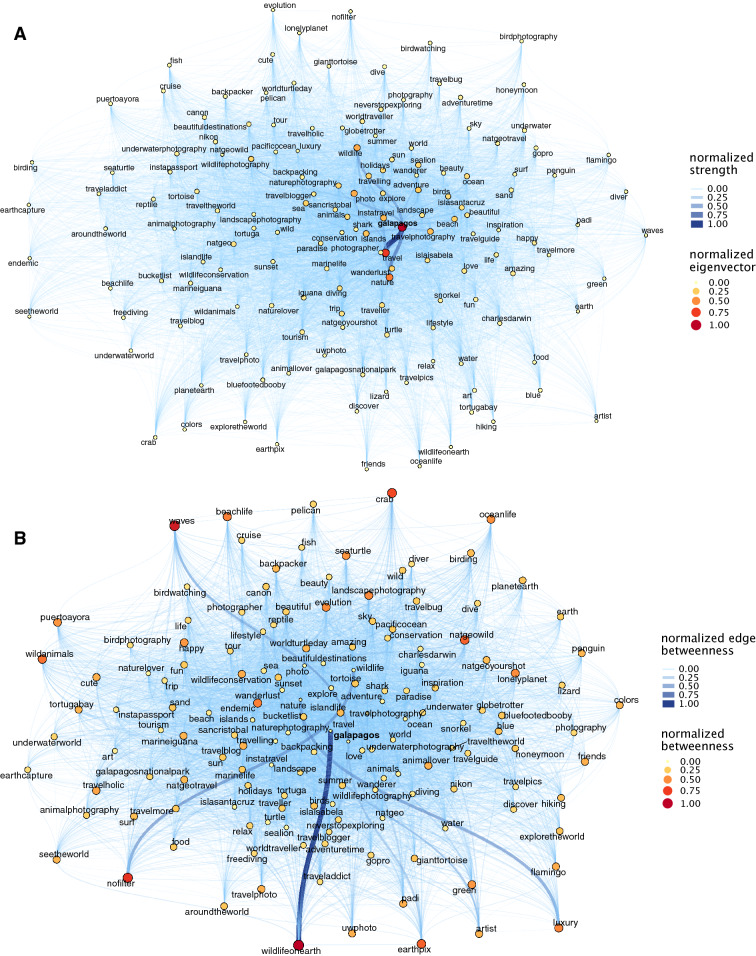
Example of network graphs in Galapagos case study. In plot (**A**) node size represents the Eigenvector centrality and edges represent normalized strength (weighted degree). In plot (**B**) node size represents normalized Betweenness centrality and edges represent normalized Edge betweenness.

Results indicated that network graphs captured information on distinct types of ecosystem services, for example, those based on wildlife and nature, heritage, or beach tourism. In areas such as Galapagos, central hashtags were *nature*, *wildlife*, *photography*, *travel* and *adventure*, evidencing a preference for wildlife and nature-based tourism. In this area, betweenness evidenced the connections between the most frequent hashtags group with other peripheric hashtags and provided a complete picture on the discourse of Galapagos’ visitors (Fig. 2). As such, nature and wildlife-based travel and photography is related with natural science concepts like evolution and endemism, and specific biotic and abiotic components like crabs and waves, altogether related with positive feelings (i.e., happy). Other areas emerging for their wildlife and nature were Skomer nature reserve, characterised by the hashtags *birds* (including the species *Puffin*), *nature* and *wildlife photography*; and Península Valdés, characterized by many locality names and by fauna, with the frequently posted hashtags’ *wildlife*, *whales* and *nature* funnelling most connections to other less frequent hashtags (e.g., *wind*, *hiking*, *relax*) and providing a full picture of the social perception on nature recreation activities, iconic fauna and positive feelings. Three networks, Sandwich Harbour, Glacier Bay and Macquarie Island also included popular hashtags related with *nature*, *wildlife* and *photography*; however, most hashtags had low betweenness and edge betweenness limiting the diversity of the posts (all network graphs are available at the Figshare repository, 10.6084/m9.figshare.13325627.v2).

Regarding cultural heritage, Easter Island was characterised by popular hashtags related with Easter Island stone statues (moais) and with travel; and edge betweenness evidenced a diversity of peripherical nodes that describe other cultural elements, like *design*, *music* and *food*, and evidence social preferences for different cultural elements of the island, beyond the moais*.* Other areas reflected cultural identity by the frequent post of local names (e.g., Ytrehvaler), words related with the country’s identity (e.g., Isole Egadi) and positive feelings about this identity (e.g., Tawharanui). In Tayrona National Park network, the full discourse identified cultural identity like *Kogui* (indigenous culture) linked with the popular posts related with nature and summer holidays. Similarly, in Tawharanui and Isole Egadi, *beach*, *nature* and *summer* where the most frequent posts that, in some cases, where connected with places and activities. In these cases, and particularly in Isole Egadi and Ytrehvaler, edge betweenness allows to identify connections between places and activities, wildlife or natural structures, providing relevant information for area management and conservation.

**Figure 3:**
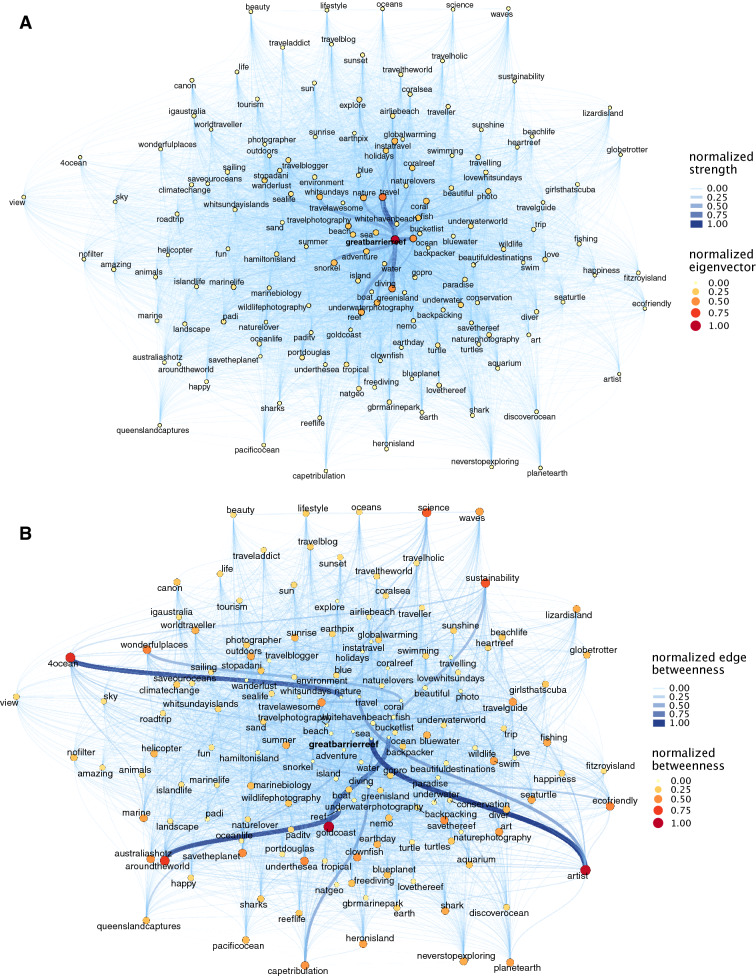
Example of network graphs in Great Barrier Reef case study. In plot (**A**) node size represents the Eigenvector centrality and edges represent normalized strength (weighted degree). In plot (**B**) node size represents normalized Betweenness centrality and edges represent normalized Edge betweenness.

A group of areas were appreciated by their underwater ecosystems. For Great Barrier Reef, popular hashtags were related with the coral reef: *ocean*, *diving*, *underwater photography*, *travel*, *nature*, *coral* and *reef*; whereas betweenness highlighted a set of hashtags related with conservation: *science*, *sustainability*, *save the reef*, *4 ocean* (Fig. 3) and evidenced the presence of a conservationist discourse in the social media. In Toguean Island network, the frequent hashtags *beach*, *wonderful* and *charming* are connected to peripherical hashtags related with the sea (e.g., *sea life*, *diving*), while in Vamizi, popular hashtags were related with high-income tourism, *private island*, *travel*, *luxury travel*, and were connected to less frequent hashtags linked to the sea, including recreational fisheries. These last two examples illustrate differences in the benefits, and beneficiaries, provided by two popular touristic destinations.

### Network communities

**Figure 4:**
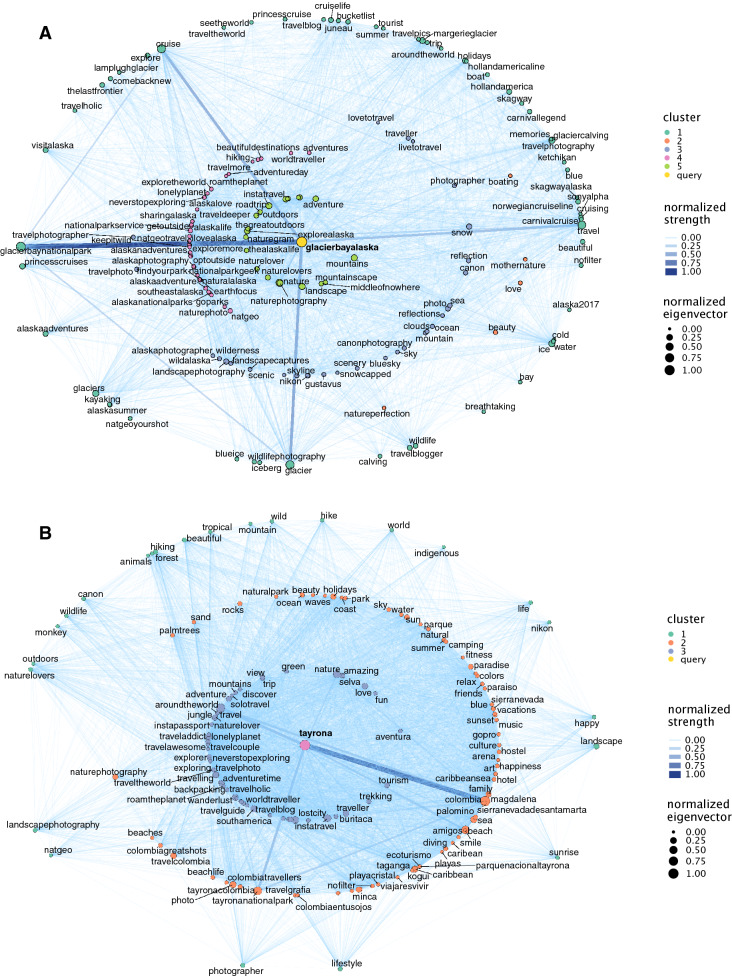
Communities assessed through Fast-Greedy algorithm for the case studies Glacier Bay (**A**) and Tayrona (**C**). The node size represents the normalized Eigenvector and the colour represents the community. The colour and width of the edges represents the normalized edge strength (weighted degree).

The division of hashtags in communities allows for a more detailed exploration of the words included in the 150 most frequent hashtags selection, independently of their centrality measures, and allowed a categorisation of hashtags within cultural ecosystem services classes in each area (Table 2). Hashtags were grouped in 3 to 5 communities, with some communities relatively constant across case studies, e.g., aesthetics, wildlife and nature appreciation (Fig. 4) (all other network graphs are available at the Figshare repository, 10.6084/m9.figshare.13325627.v2).

**Table 2:** Cultural Ecosystem Services’ types (CES) depicted from the community analysis (Fast Greedy algorithm). The order of the CES class does not imply a priority rank.

	CES 1	CES 2	CES 3	CES 4	CES 5
Galapagos	Nature and wildlife appreciation	Recreational (beach)	Other (travel)	Underwater wildlife and recreational (underwater)	Aesthetic and wellbeing
Glacier Bay	Aesthetic and nature appreciation	Aesthetic	Recreational (hiking)	Other (National Park and Glaciers)	
GBR	Underwater wildlife and recreational (underwater)	Other (travel)	Aesthetic and nature appreciation		
Isole Egadi	Recreational (water activities)	Aesthetic and wellbeing	Cultural identity	Other (travel)	
Macquarie Island	Nature and wildlife appreciation	Wildlife and conservation	Recreational and wildlife (iconic fauna)	Wildlife (bird watching)	
Peninsula Valdez	Wildlife (sea life) and recreation	Wildlife conservation	Aesthetics and recreational	Wildlife (iconic fauna)	
Easter Island	Cultural heritage	Other (adventure and travel)	Nature, aesthetics and wellbeing	Recreational (underwater)	
Sandwich Harbour	Aesthetics	Wildlife, aesthetics and recreational	Wellbeing and recreational (safari)		
Skomer	Aesthetic and recreation (hiking)	Wildlife (birds) watching	Wildlife (birds)		
Tawharanui	Recreational (beach)	Nature, aesthetic and wellbeing	Cultural identity	Wildlife conservation	
Tayrona	Wellbeing and aesthetics	Recreational (hiking) and cultural heritage	Nature and aesthetics		
Togean Island	Other (travel)	Underwater wildlife and recreational (underwater)	Aesthetics, wildlife (underwater) and recreational (underwater)		
Vamizi	Nature, wildlife and conservation	Recreational (underwater) and other (luxury tourism)	Aesthetics and wellbeing	Recreational (fishing)	
Ytrehvaler	Nature and cultural identity	Nature and recreational (hiking and kayak)	Recreational (hiking)	Nature and aesthetics

In some of the areas, the communities were diverse in hashtag composition, for example, in Galapagos, *wildlife* (and related words) was distinctive of several communities, but other communities were characterised by different concepts: *beach*, *holidays*, *happiness*, *snorkelling* and *diving*. In Easter Island, the hashtags related with the stone statues and cultural heritage characterise one community, while the other communities include a diversity of hashtags classified under adventure, nature, underwater recreational activities; therefore, it widens the information provided by the centrality metrics. Tayrona (Fig. 4) is also a diverse network with one community characterised by hashtags like *beach*, *summer*, *happiness* (wellbeing), but other communities contain a diversity of hashtags like *forest*, *hiking*, *indigenous* and *wildlife* (classified in recreational, cultural heritage, nature and aesthetics; Table 2).

In some areas, the communities were not so diverse, but provided additional information on the posts. For example, in MacQuarie Island the communities highlighted iconic fauna, including several penguin species, and biodiversity conservation. In several areas, network communities informed of the iconic fauna and specific places: puffins and other bird species in Skomer; southern right whale, sealions and penguins in Península Valdés; glaciers and mountains in Glacier bay (Fig. 4); desert and dunes in Sandwich harbour. Finally, Ytrehvaler is a network characterised by many local names (in Norwegian), evidencing a national tourism, and hashtags related with scenery.

### Merged network of the 14 case studies

**Figure 5:**
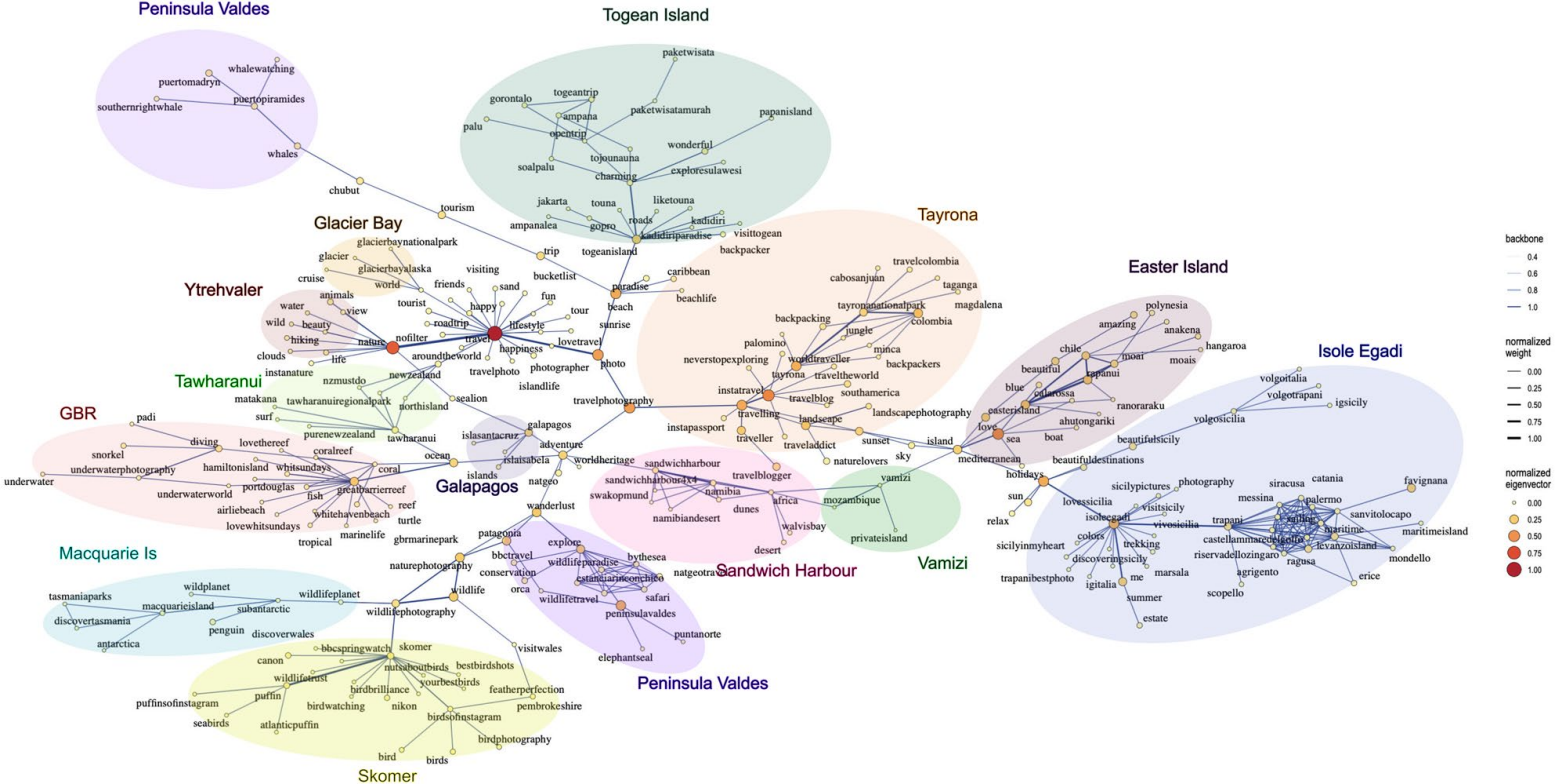
Global network graph including the fourteen case studies where the node size represents the Eigenvector centrality. The coloured clusters arrange the case studies to facilitate the visual identification of areas connected in the network.

The merged network highlighted several hashtags that act as bridges between communities of hashtags (Fig. 5). *Nature*, *travel*, *photo* and *travel photography* are key to structure the global network. However, several low eigenvector hashtags connect smaller groups: *sunset* and *island* connect the subgroups from Easter Island, Isole Egadi and Vamizi.

From the hashtag *travel photography* diverges a branch that connects 7 areas through *adventure*; a small group of hashtags deriving from this node represent Sandwich harbour and Vamizi, connected through *Africa*. The hashtag *ocean*, connected to *adventure,* relates Great Barrier Reef with Tawharanui, and to *wanderlust* (a German expression for the desire to explore the world) that connects Península Valdés, Skomer and Macquairie Island. These three areas and Tayrona are also connected through the central hashtag *travel photography*, and Skomer and Macquairie Island *through wildlife photography*. The hashtag *adventure* is also connected to a group of hashtags from Galapagos that also derive to the high eigenvector hashtag *nature*.

The hashtag *nature* is key to include the fragile sub-network Ytrehvaler, and also derives to other high eigenvector hashtag, *travel*, that in turn, connects to the small sub-network from Glacier bay. *Photo,* a central hashtag related with *travel,* connects to *paradise*, that is key to integrate Toguean Island, a few hashtags from Tayrona related with the Caribbean and beach, and a group of hashtags from Peninsula Valdez related with whale watching. Some other small hashtags, that are connected to high eigenvector hashtags but are not included in any particular area are shared by many of the areas, e.g., *sun*, *relax*, *landscape photography*, *nature lovers*, *sunset*, *sky*.

## Discussion

The analysis of social media data with Graph Theory provides a powerful indirect approach to monitor visitors’ preferences and perceptions for marine and coastal areas, and it provides a global view of the wide array of benefits humans obtain from nature. The analysis of users’ posts with different network centrality metrics allowed the identification of an emergent discourse in Instagram by identifying the most frequent words related with a specific area (e.g., *nature*, *wildlife* and *photography* in Galapagos), and also other less frequent words (e.g., evolution and endemism), including feelings (e.g., *happy*), connected with the most frequent words. The primary and secondary information provided by the centrality metrics is of high value for conservationists and managers, as it characterises visitors’ preferences, including recreational activities and preferred ecosystems and species. Importantly, our approach allowed to gather this individualised information remotely from a wide variety of marine and coastal case studies around the world.

What becomes evident from this global assessment is that an area does not need to be an iconic destination to contribute to society’s recreation and wellbeing. Galapagos, Great Barrier Reef or Easter Island provide essential services like nature appreciation, wildlife watching or cultural identity, however, such services have also been identified in less iconic areas often visited by local tourists like Skomer Island, Tawharanui and Ytrehvaler. Similarly, wellbeing related with relaxation and happiness is recorded in remote and iconic areas like Galapagos, but also in less internationally known places such as Vamizi, Tawharanui or Isole Egadi. The frequent post of the word *happiness* (and similar words) denotes the importance of nature’s contribution to people’s wellbeing, as emphasised by several authors^33–35^, with many benefits arising from human connections with nature, including sense of place, identity, mental health and sense of belonging. These benefits were independent of its location, ecosystem or main activity provided. A series of cultural ecosystem services’ bundles could be identified in each case study, evidencing areas with high diversity of benefits and perceptions, whereas other areas were relatively homogenous in users’ activities and perceptions. Frequent words’ groups were related with aesthetics, wildlife and nature appreciation, which is expected as information is obtained from a photography-based social media platform. However, the classification of the popular hashtags in cultural ecosystem services’ types, despite providing standardised information that allows the comparison with other studies, limits the information depicted from the networks. Network analysis allowed moving beyond the state of the art by mere hashtag frequency to the exploration of the nature of connections between hashtags, delineating the users’ discourse. For example, animal species connected to local names provides information on places for wildlife watching, e.g., puffins in Skomer Island, or penguin species in MacQuarie Island. Hashtags also evidenced environmental awareness, e.g., conservation in Macquarie Island or climate change in Great Barrier Reef, which should be considered key to promote transformative changes for policy makers^36^. In summary, relevant words like nature watching can be linked to a place or to a species name, conservation can be linked to a place or ecosystem component, and so on.

Instagram provides information on what calls the attention of visitors, but also on activities and feelings about the place. For example, many Instagram users mentioned natural spaces for their conservation value (e.g., Great Barrier Reef), nature excursions (e.g., Glacier Bay), bird watching (e.g., Skomer), views of natural fauna (e.g., Península Valdés), but also, and not least, the scenic beauty (e.g., Sandwich harbour), the relaxation and happiness provided by open spaces (e.g., Galapagos), or some luxury accommodations within national parks as wellness spaces (e.g., Vamizi). The main activities reported in each case study were generally related with the dominant habitat, e.g., diving in Great Barrier Reef or Toguean Island, with prevalence of coral reefs, or beach recreation in Tawharanui or Isole Egadi, characterized by sandy beaches. Nevertheless, the posts’ discourse in each area is markedly defined by the visitor profile, and access to the area. For example, Vamizi is characterized by high-income and international tourism that visit the area for the opportunity to enjoy the underwater life, recreational fishing and beach. Other areas like Skomer and Ytrehvaler are mainly visited by locals that enjoy the wildlife and nature of the place. This implies that enjoyment of natural spaces by people is highly conditioned by its logistical accessibility, with remote places like Macquarie Island or Galapagos visited by international tourists that travel (*travel*, being one of the most common hashtags) to these areas to find adventure, recreation, or scenery, amongst others. More accessible areas (i.e., those easily accessible from large cities), like Tawharanui, Ytrehvaler or Isole Egadi, appear to be predominantly visited by locals in search of the relaxation of the beach, sighting of emblematic local fauna, or nature recreation. The merged network evidenced that central hashtags to all areas were *nature*, *travel*, *photo*, however, less popular hashtags appear key to connect smaller groups of areas and were related with general concepts posted in these areas and not with principal activities or focus. For example, *sunset* and *island* connected Easter Island, Isole Egadi and Vamizi, *wanderlust* connects Península Valdés, Skomer and Macquairie Island, *paradise* connects Toguean Island and Tayrona, *ocean* connects Great Barrier Reef and Tawharanui. These results imply that the perception of visitors to the areas is not exclusively conditioned by the main activity or ecosystem type, but by higher level concepts like *paradise* or *wanderlust*.

It has been acknowledged that the use of information from social media platforms has an inherent bias associated to both the type of user and the type of content posted on the platform. Hausmann et al.^21^ observed that while most of the pictures posted on Flickr focused on biodiversity, Instagram was popular for sharing pictures about activities and people. On the other hand, Ruiz-Frau et al.^27^ observed that Twitter posts reflected social awareness and discussions around current global concerns such as climate change and youth movements. Therefore, different social media platforms may be used by different groups of people. Aiming to maximize the representation of the wider society, we considered Instagram to be an optimal platform for representing a wide diversity of people and of natural spaces. However, the use of this methodology is not exempt of challenges. When using social media data as a proxy for peoples’ perceptions, there is an inevitable bias towards aesthetic values^37^ and, in the particular case of Instagram, a strong dominance of content related with social recreation. Ultimately, photographs tend to express pleasant and beautiful features^38^ and Instagram is not an exception as in most case studies only positive feelings were reported (overly represented by the hashtag #happy and synonyms), with few exceptions where conservation awareness was identified in the social media discourse. Representativeness can also be a challenge^39,40^, and Instagram is mostly representing the younger generations^41^. Perceptions from people that do not post on Instagram, remarkedly from older generations, people with limited or no access to technology or people from countries where Instagram is not sufficiently dominant are not adequately represented in our approach. Nationality is also relevant, as local visitors’ perceptions can differ from international tourists’, represented by English as the vehicular language^15^. This can be partially solved by using words in different languages as queries, like for Easter Island (e.g., Rapanui, Isla de Pascua and Easter Island), or by including words in different languages in the network, like in the Norwegian reserve of Ytrehvaler. However, social media platforms are sometimes restricted in certain countries and it might imply an important bias in the nationality of tourists encompassed in the analysis.

Additional challenges are linked to the extraction of data from Instagram. Results showed that a manageable sample of posts can provide valuable information about peoples’ perceptions for a natural area. Nevertheless, a sufficient volume of posts might not be available for particular areas. In addition, the application of this methodology is restricted to those areas with a unique name to be used as a query in order to avoid downloading information from other areas which might have the same name. Since June 2020, access restrictions to Instagram data have incremented and our approach could not be currently replicated without incurring into web scrapping. Nevertheless, the authors support the use of the approach for scientific purposes and defend access to big data stored in social media after request. Despite these limitations, our study approach provides many advantages, including (1) cost and effort effectiveness in data collection and processing, (2) remote collection of information that allows large scale studies and (3) minimization of researchers’ subjectivity through the use of centrality metrics to explore the emerging properties of a network of words. Moreover, while the assessment of visitors’ perceptions in natural spaces is generally conducted during peak visitation season and restricted to frequently visited locations^10^, the remote collection of social media data can encompass any temporal dimension, and, in principle, it covers visitors to all locations within the natural areas. The variability in visitors’ preferences can assist managers and policy makers design tailored strategies to promote nature conservation for visitors’ enjoyment, which is of high relevance when destination sites are often ecologically or culturally fragile^23,42^. The continuous low cost-effective monitoring of social media can allow a better understanding of spatial–temporal changes in visitor preferences^21^, and this approach can now materialise with the prevalence of smartphones and the posting of experiences in social media facilitating the remote access to large scale information on peoples’ perceptions and use of natural spaces. In the internet era, with ever-increasing amounts of data stored in web-based platforms, access to this data with scientific purposes is crucial. We encourage the scientific community to claim access, while accepting restrictions to personal or sensitive data.

## Conclusions

The emergent properties of networks of Instagram hashtags were explored with Graph Theory to characterise the array of interactions between nature and people using a cultural ecosystem services lens. The information obtained includes visitors’ preferences (e.g., cultural heritage, wildlife and nature appreciation), but also activities (e.g., diving, hiking, relaxing), preferred habitats or species (e.g., forest, beach, penguins), and feelings (e.g., happiness, beach lifestyle, place identity). Our approach allowed to identify places valued for their cultural heritage (e.g., Easter Island’s moais), but also for their iconic species (e.g., puffins in Skomer island) or natural monuments (e.g., sand dunes in Sandwich harbour), and sense of place and identity (e.g., Isole Egadi and Tawharanui). We introduce a tool that has allowed us to explore peoples’ perceptions from a wide diversity of marine and coastal areas globally. Importantly, this approach allows to identify and monitor the variable benefits people obtain from nature in a cost-effective but holistic way, essential for the effective conservation of natural species.

## Methods

### Case studies

Fourteen marine and coastal areas were chosen for the study in order to encompass a wide diversity of marine and coastal ecosystems across regions (Table 1). The selected areas had to comply with two criteria: (1) the area had to be sufficiently popular to contain enough data for the analysis; (2) the name of the area had to be sufficiently characteristic to provide a unique alphabetical identifier within Instagram, without the need to use filters or metadata allowing the geolocation of the photos. The adoption of these criteria meant that no area in the Arctic (marine realm 1) containing enough data could be identified in Instagram. Some of the realms established by Spalding et al. (2007) are too broad to capture existent variability across systems (e.g., temperate Northern Atlantic and Mediterranean Sea); when the authors considered this was the case, more than one study area were selected to capture this variability (Fig. 1).

### Social media data

Data collection and analysis were carried out according to the methodology established in Ruiz-Frau et al.^27^. Instagram posts were downloaded through the Application Programming Interface (API), with a specific development for the R language and environment for statistical computing version 3.6.0, released 2019-04-26 (R Development Core Team, 2009). Data download was completed in June 2019; since June 2020, the Instagram API increased restrictions impeding access to the stored data through our R code. In consequence, the methodology described here cannot be directly replicated. A javascript procedure could be used to fetch data from a loop through the posts; however, this simple procedure could be considered webscrapping and may infringe the intellectual property rights. Nevertheless, the authors support the use of the approach for scientific purposes and defend access to big data stored in social media after request.

The Instagram API was suitable for a hashtag-based data extraction using a creator account. Post retrieval was done according to the platform internal algorithms. The authors of this study did not have complete access to the algorithms’ descriptions, and, while API search appears to be chronological, the company's public documentation does not provide specific information to confirm this. For privacy and ethical reasons, no personal information, such as user’s names or ids, were used in this study. Likewise, the metadata of the photographs or the geolocation information that some users add to their posts was never accessed. Results are presented in an aggregate manner so that no information can be traced back to the individual user (more details of the methods in Ruiz-Frau et al.^27^).

For each case study, a search query was executed (Table 1). We aimed to download 10,000 posts per case study in June 2019. The data download started with the most recent post and was followed by the previous post until reaching the cut-off (i.e., 10,000). Frequently, the areas had fewer than the established threshold (i.e., 10,000 posts) and in such cases we downloaded all available posts, never bellow 1000 posts (Table 1). Downloaded posts for each case study were stored locally and datasets were filtered and cleaned in order to retain only relevant information for further analysis^43,44^. Posts often contain non-relevant information as social media platforms are frequently used as marketing and advertisement tools to reach a wider public and often bots (automated data generating algorithms and advertisements) are used to create large volumes of automated posts. Automatically generated posts were detected using graph based^45^, anomaly detection^46,47^, and time series analysis^48,49^ approaches. Most of the time, the use of a graph-based approach was sufficient to eliminate almost all of the posts generated by bots, thus making the use of anomaly detection and time series analysis circumstantial. Additionally, irrelevant posts, mostly related to human-generated advertising (e.g., human-written posts related with a trading mark named Galapagos or Rapanui, unrelated with the natural capital of the areas), were detected by a simple visual inspection of a preliminary graph and discarded for further analysis. Discarded posts were selected by specific hashtags (e.g., #chocolate, frequently linked with #rapanui due to a trademark) or a specific user (i.e., those users identified as posting irrelevant marketing). The number of discarded posts never exceeded 25% of the total number of downloaded posts (e.g., 2500 discarded posts when 10,000 posts were downloaded). Dataset cleaning also consisted in merging similar words (e.g., #travelgram, #instatravel, #igtravel) and misspellings (e.g., #travel, #travell). Highly frequent non-English words were translated to English (e.g., #statue, #steinfigure, #estatua; for Easter Island statue in different languages) to homogenise the network language and avoid numerous duplicates. However, in some networks with a prevalence of non-English language (e.g., Ytrehvaler in Norwegian) words were not translated to English to capture users’ characteristics.

### Graph Theory

In this study, we apply network graph visualization tools to the hashtags associated to Instagram posts. The hashtag is represented by a node, with the size of the node representing the relevance of the hashtag, and the relationship between each pair of nodes is identified with a link (edge). The width of the edge represents the strength of this connection. Centrality measures are useful to determine the relative importance of nodes and edges within the overall network^28^. In networks consisting of several nodes, some of them play a decisive role in facilitating a large number of network connections. Such nodes are central in network organization and are often identified by a range of metrics known as centrality measures. The identification of important, or *central,* vertices in a network is a key aspect in the definition and description of networks^50^. However, there are multiple interpretations of what makes a node important and there are therefore many measures of centrality^28^. We calculated different centrality measures and, after a first screening including 11 measured of centrality for the Instagram hashtags’ network (e.g., Degree, In-Degree, Out-Degree, Strength, HubScore; see Ospina-Alvarez et al.^51^ for a detailed description of the different measures), we selected *betweenness* and *eigenvector centrality* to illustrate and interpret the structure of the social networks.

Eigenvector centrality measure^52^ takes into consideration not only how many connections a node has, but also the centrality of the vertices that it is connected to. Eigenvector centrality ranks higher those vertices that are connected to important neighbours, i.e., other vertices that are connected to many other well-connected vertices. It is a measure of the influence of a node in a network. In our study context, hashtags with high eigenvector values are high frequency hashtags that in turn are connected to other high frequency hashtags, and so on.

Betweenness centrality^28^ is a measure of the influence of a node over the flow of information between every pair of vertices under the assumption that information primarily flows over the shortest paths between them. Betweenness centrality indicates nodes that have a high probability of having routes that connect them to other nodes in the network. Similarly, edge betweenness centrality is defined as the number of the shortest paths that go through an edge in a graph or network^53^. Each edge in the network can be associated with an edge betweenness centrality value. An edge with a high edge betweenness centrality score represents a bridge-like connector between two parts of a network, the removal of which may affect the communication between many pairs of nodes through the shortest paths between them. In our context, betweenness centrality and edge betweenness centrality provide information about clusters of hashtags that describe users' experiences or perceptions and that connect (by means of a hashtag) to other clusters representing other types of experiences or perceptions. The removal of high Betweenness hashtags would fragment the network and disconnect distant concepts.

### Data analysis

The first 150 hashtags (frequency > 1.5% when 10,000 posts are retrieved) had a probability of more than 90% of occurring with any other of the first 150 hashtags in the same post. Therefore, this criterion was used to create networks with great cohesion and connectedness, representing the dominant discourse in relation to the area in question. Network graphs were delineated using eigenvector, betweenness and edge betweenness as centrality measures.

In order to find any evidence for an emergent pattern of clustering among the hashtags for each of the 14 case studies, hashtags were assigned to network communities through the use of Fast-Greedy community algorithm^27^. Fast-Greedy algorithm makes the best choice at each small step in the hope that each of these small steps will lead to a globally optimal solution^54^.

In order to visualise potential similarities in the social media discourse across the 14 case studies, all the data was merged, and the 1400 most frequent hashtags pairs were retained for analysis in a single network graph. Similar to what was previously described for the individual networks, these 1400 pairs of hashtags accounted for over 90% of the linkages between hashtags, representing the dominant discourse across the 14 areas. Eigenvector centrality was used as the measure of node influence in the merged network and connections were represented with a backbone layout^55^. This layout has proven effective to illustrate networks with most vertices in a central position that result in high overlap in large networks^56^.

We used the open source graphics manipulation software *igraph*^57^ to obtain the centrality measures and communities aggregations. Graphics and figures were generated using the visualization software *ggraph*^58^ and *ggtree*^59^. All of the above software can be used as extension packages of the R language and environment for statistical computing (R Development Core Team, 2009) freely available online.

## Data Availability

All the network figures generated during the current study are available in the Figshare repository, 10.6084/m9.figshare.13325627.v2.
